# Monoclonal Antibodies Recognizing the Surface Autolysin IspC of *Listeria monocytogenes* Serotype 4b: Epitope Localization, Kinetic Characterization, and Cross-Reaction Studies

**DOI:** 10.1371/journal.pone.0055098

**Published:** 2013-02-04

**Authors:** Jennifer Ronholm, Henk van Faassen, Roger MacKenzie, Zhiyi Zhang, Xudong Cao, Min Lin

**Affiliations:** 1 Ottawa Laboratory Fallowfield, Canadian Food Inspection Agency, Ottawa, Ontario, Canada; 2 Department of Biochemistry, Microbiology and Immunology, University of Ottawa, Ottawa, Ontario, Canada; 3 Institute for Biological Sciences, National Research Council Canada, Ottawa, Ontario, Canada; 4 School of Environmental Sciences, University of Guelph, Guelph, Ontario, Canada; 5 Institute for Microstructural Science, National Research Council Canada, Ottawa, Ontario, Canada; 6 Department of Chemical and Biological Engineering, University of Ottawa, Ottawa, Ontario, Canada; Wadsworth Center, New York State Department of Health, United States of America

## Abstract

*Listeria monocytogenes* serotype 4b is responsible for a high percentage of fatal cases of food-borne infection. In a previous study, we created 15 monoclonal antibodies (MAbs) against a ∼77 kDa antigen that is associated with the cell surface of live *L. monocytogenes* serotype 4b cells. Here we report an extensive characterization of these MAbs to further their development as diagnostic reagents. The ∼77 kDa target antigen was identified by mass spectrometry and N-terminal sequencing to be IspC, a novel surface associated autolysin. Epitope localization experiments revealed that each of the 15 MAbs recognized the C-terminal cell-wall binding domain of IspC. The presence of IspC was shown to be highly conserved within *L. monocytogenes* serotype 4b, as evidenced by a strong reaction between anti-IspC MAbs and all 4b isolates. To determine the range of cross-reactivity with other *L. monocytogenes* serotypes ELISA was used to test each MAb against multiple isolates from each of the *L. monocytogenes* serotypes. Of the 15 MAbs, five: M2774, M2775, M2780, M2790 and M2797, showed specificity for *L. monocytogenes* serotype 4b and only cross reacted with serotype 4ab isolates. The kinetics of the interaction between each of the MAbs and IspC was measured using surface plasmon resonance. The MAbs M2773, M2792, M2775, M2797 and M2781 each had very low dissociation constants (4.5 × 10^−9^ to 1.2 × 10^−8^ M). While several of these antibodies have properties which could be useful in diagnostic tests, the combined high fidelity and affinity of M2775 for the IspC protein and serotype 4b isolates, makes it a particularly promising candidate for use in the development of a specific *L. monocytogenes* serotype 4b diagnostic test.

## Introduction

*Listeria monocytogenes* is a facultative, intracellular, bacterial pathogen that is the etiological agent of listeriosis. Infection with *L. monocytogenes* is of particular risk for certain demographics including: neonates, pregnant women, the elderly and those with impaired T-cell mediated immunity such as HIV or transplant patients, since listeriosis in these individuals is associated with extremely high fatality rates. *L. monocytogenes* is frequently found in the environment, can multiply at refrigeration temperatures, and is able to survive in a wide range of salt concentrations, temperatures and pH conditions making its presence in food processing plants difficult to control [1]. Therefore *L. monocytogenes* is a concern for the Ready-To-Eat (RTE) food-processing industry, since RTE products are consumed directly without cooking.

*L. monocytogenes* is divided into 13 serotypes, of which only 3 serotypes (1/2a, 1/2b and 4b) are associated with the majority of human illnesses [2]. Serotype 4b isolates are of particular importance, given that they are responsible for more cases of listeriosis than serotype 1/2a and 1/2b isolates combined, despite serotype 1/2a and 1/2b strains having a higher prevalence in foods and the environment [3], [4]. Serotype 4b strains are more commonly isolated from patients suffering from meningoencephalitis than from patients with infection limited to the blood-stream [3]. Listeriosis patients suffer a high mortality rate of 26% when infected with a serotype 4b strain compared to the rate of 16% for patients infected with a serotype 1/2a or 1/2b strain [5]. This epidemiological data suggests that serotype 4b strains may be more adapted to and therefore more virulent in human hosts than other serotypes [3], [4]. Current regulatory standards do not differentiate between serotypes and recent outbreaks caused by non-serotype 4b strains indicate the importance of the zero-tolerance policy for *L. monocytogenes* in foods. However, the development of a diagnostic reagent specific for *L. monocytogenes* serotype 4b strains would be helpful in monitoring and surveillance of this particularly important serotype.

Current culture based methods for detecting *L. monocytogenes* are labour intensive and take 5–7 days for detection and serotyping. Molecular techniques such as PCR are very valuable as a rapid detection step to reduce the turnaround time from sampling to test results; however, they face the challenge and obstacle of a lengthy sample preparation time, which sometimes includes culture enrichment to increase the number of target pathogen prior to detection. In addition, if PCR is preformed without culture enrichment there is no way to determine if the contaminant bacteria are viable. These inherent drawbacks have prompted us to seek methods for rapid isolation and detection of *L. monocytogenes* serotype 4b. Antibody-based methods have been demonstrated to be very promising for rapid isolation and detection of *L. monocytogenes* from food and environmental samples [6], [7]. Antibodies with certain binding characteristics, such as a high affinity and specificity for a surface localized protein of *L. monocytogenes* make them especially useful for bacterial isolation and detection by immunological methods. With such high quality antibodies in conjunction with the advent of new technologies, cultural enrichment may not be necessary for detection. Currently the monoclonal antibody (MAb) used in the VIDAS LMO assay (bio-Merieux, Marcy-Etoile, France), can discriminate between *L. monocytogenes* and other species of *Listeria* making it a useful screening tool in food pathogen testing laboratories [8]. Attempts to develop MAbs against *L. monocytogenes* serotype 4b have been made [9]; however, results with their use were inconsistent and the surface markers recognized by those MAbs remained unidentified and not characterized. Therefore, there is currently no antibody available to specifically detect *L. monocytogenes* serotype 4b.

Our lab has generated 29 MAbs against *L. monocytogenes* serotype 4b [10]. Of these MAbs, 13 did not recognize linear protein epitopes and were not investigated further, however, 16 recognised a protein with an apparent molecular weight (MW) of 77 kDa and were able to bind to the surface of both live and formalin-killed *L. monocytogenes* cells [10]. In this study, we aim to determine the molecular identity of the antigens recognized by these MAbs, localize the epitopes within the antigen, define the kinetic properties of the MAb-antigen interaction and examine cross-reactivity with a wide range of *L. monocytogenes* isolates representing various serotypes. The results show that the immunogenic surface protein C (IspC) (Genbank access no. EF409982), an autolysin with N-acetylglycosaminidase activity [11], is recognized by this group of MAbs and is conserved in serotype 4b strains. IspC is an extremely promising target for the detection of *L. monocytogenes* serotype 4b.

## Materials and Methods

### Bacterial Culture

*L. monocytogenes* isolates included in this study (Table S1) were grown in Brain Heart Infusion (BHI) broth or on BHI agar plates (BD Biosciences, Mississauga, ON) at 37°C and cell concentrations were estimated as previously described [12]. *Escherichia coli* stains (DH5α and Rosetta DE3/(pLysS)) used in this study were cultured in Luria-Bertani (LB) media (BD Biosciences) supplemented with 50 µg/mL kanamycin as required.

### Extraction of Listeria Surface Proteins

*L. monocytogenes* strain LI0521 (serotype 4b) was grown to stationary phase high density in BHI broth and non-covalently bound surface antigens were released from the cells by boiling 8.7×10^10^ cells in 20 mL PBS containing 4% (w/v) sodium dodecyl sulphate (SDS) for 10 min. After cell suspensions were centrifuged at 10000 × g for 10 min the surface proteins were contained in the supernatant. SDS was removed from the supernatant by chromatography with Extracti-Gel D Detergent Removing Resin, according to the manufacturer’s instructions (Thermoscientific, Rockford, IL, USA). The concentration of surface proteins was determined by the Bradford Protein Assay using Bio-Rad Protein Assay Dye Reagent Concentrate (Bio-Rad, Mississauga, ON, Canada), according to the manufacturer’s instructions, using bovine serum albumin (BSA) as a standard.

### SDS-PAGE and Western Blotting

SDS-polyacrylamide gel electrophoresis (SDS-PAGE) was performed as described by Laemmli [13], using a 4% stacking gel and a 12% resolving gel with the Bio-Rad minigel apparatus. Separated proteins were visualized in the gel using Coomassie blue staining, or by western blotting with MAbs using a 1∶50 dilution of tissue culture fluid (TCF) in PBS containing 3% (w/v) bovine serum albumin (BSA). Bound antibodies were detected using a 1∶2000 dilution of Peroxidase-AffiniPure Goat Anti-Mouse IgG (Jackson ImmunoResearch, West Grove, PA, USA) in PBS containing 3% BSA and the horseradish peroxidase (HRP) substrate kit (Bio-Rad, Mississauga, ON, Canada).

### Immunoprecipiation

The M2799 MAb was selected for immunoprecipiation because of a strong and specific reaction with the antigen as judged by western blotting. M2799 was purified from TCF by affinity chromatography, on a Protein G Sepharose 4 Fast Flow column (GE Healthcare, Baie d’Urfe, QC, Canada), prior to its use in immunoprecipitation. Twenty µg of Protein G purified M2799 was combined with 26 µg of extracted surface protein in PBS and a cOmplete EDTA-free protease inhibitor Cocktail Tablet, used according to the manufacturer’s instructions (Roche Canada, Mississauga, ON, Canada). The protein mix was then incubated at 4°C for 2 hrs with constant agitation. Protein A sepharose 4B beads (150 µl) (Invitrogen, Burlington, ON, Canada) were washed with PBS prior to use and added to the MAb-surface protein mix and incubation continued at 4°C for an additional 4 hrs. Beads were collected by centrifugation and washed multiple times in PBS containing a protease inhibitor cocktail before being suspended in 2× SDS-PAGE loading buffer and boiled for 10 min. The proteins released into the supernatant were separated by SDS-PAGE and protein bands were visualized by Coomassie blue staining. The ∼77 kDa antigen was excised from the gel and sent to the Ottawa Hospital Research Institute (OHRI) Proteomics Facility (Ottawa, ON, Canada) for protein identification by mass spectrometry (MS). In addition, the separated 77 kDa protein was also blotted onto a polyvinylidine fluoride membrane, excised after staining with Coomassie blue, and sent to the protein core facility at Columbia University College of Physicians and Surgeons (New York, NY, USA) for N-terminal Edman sequencing.

### Expression Constructs for IspC and Truncated Fragments

DNA manipulations were performed according to previously established procedures [14]. Fragments of the *ispC* ORF were amplified from pIspC [15] DNA by PCR with the primers listed in Table S2 and ligated into the *Nde*I and *Not*I sites of a double-digested pET-30a (Novagen, Madison, WI, USA). Isopropyl-β-D-thiogalactopyranoside (IPTG) (1 mM) was added to induce expression of the recombinant proteins for 3 hrs at 37°C and then at 4°C for 16–18 hrs. Expression of each IspC fragment was verified by SDS-PAGE followed by western blotting with a Penta-His Antibody (Qiagen). For epitope localization, the IspC fragments were analyzed by western blotting probed with selected MAbs at 1∶50 dilution of TCF.

### Expression and Purification of Recombinant IspC

Recombinant IspC (rIspC), expressed from the construct pIspC in *E. coli* Rosetta (DE3)/pLysS cells, was purified essentially as described in Wang and Lin [15], with some modifications. Briefly, an overnight culture was diluted 1∶100 in LB containing kanamycin (50 µg/mL) and subcultured until an OD_590_ of 0.6±0.1 was reached. IPTG (1 mM) was added and the culture was incubated at 37°C for 4 hrs and then at 4°C overnight. Soluble recombinant IspC was purified by a combination of metal chelate affinity chromatography using Ni-NTA Superflow (Qiagen), followed by cation-exchange chromatography on a column of SP Sepharose Fast Flow (GE Healthcare). To reach the purity required for future analysis an additional step, size exclusion chromatography using a Superdex™ 200 column (GE Healthcare) under the control of an AKTA™ fast protein liquid chromatography (FPLC), was performed.

### Preparation of Antibody Fab Fragments

MAbs were purified from TCF by affinity chromatography using a column of CNBr-activated Sepharose 4B (GE Healthcare), conjugated with the recombinant IspC according to the manufacturer’s instructions. Purified anti-IspC MAbs were digested with papain (Sigma, Oakville, ON, Canada) in 100 mM glycine-HCl, pH 7.0, 100 mM dithiotheitol, and 50 mM EDTA, where the amount of papain was 1% that of MAb, for 2 hrs at room temperature. The digestion reaction was quenched with 10 mM iodoacetamide. Complete digestion of the MAbs was verified by SDS-PAGE. The digested antibodies were dialyzed overnight into 20 mM HEPES buffer, and run on a Protein G column to remove the Fc fragment from the Fab fragment. The column flow through, containing the Fab, was analyzed to ensure it was free of Fc fragment by SDS-PAGE followed by western blotting using an Fc specific antibody (Jackson ImmunoResearch). The Fab flow through was concentrated using an Amicon Ultra-15 centrifugal unit (10 kDa MWCO) (Millipore, Billerica, MA, USA). Fab fragments were stored in Glycine-Tris-HCl at pH 7 with 0.05% sodium azide at 4°C. Size exclusion chromatography was performed on all Fab samples, immediately prior to surface plasmon resonance (SPR) analysis, using a Superdex™ 75 column (GE Healthcare) controlled by an AKTA™ FPLC. Only the peak fraction from the size exclusion column was used in affinity measurements.

### Surface Plasmon Resonance Analysis

The binding of Fab fragments to immobilized IspC was determined by SPR using the BIACORE 3000 (GE Healthcare). A total of 1016 resonance units (RU) of IspC were immobilized on a research grade CM5 sensor chip. The immobilization was carried out using 50 µg/mL IspC in 10 mM acetate at pH 4.0 using the amine coupling kit which was supplied by the manufacturer. An ethanolamine blocked surface was used as a reference. The affinity measurements were carried out at 25°C in HBS-EP running buffer (10 mM HEPES, pH 7.4 containing 150 mM NaCl, 3 mM EDTA and 0.005% surfactant P20). A flow rate of 20 µl/min was used and Fab sample volumes were 200 µl giving a 10 min injection time followed by a 10 min dissociation time. Surfaces were regenerated by washing with either HBS-EP running buffer (M2774, M2779, M2788, and M2790) or 10 mM glycine pH 2.0 for the other Fabs. Data were analyzed with BIAevaluation 4.1 software.

### Indirect ELISA

The MAbs were assessed for cross-reactivity with a variety of *L. monocytogenes* isolates, including serotypes 1/2a, 1/2b, 1/2c, 3a, 3b, 3c, 4a, 4b, 4ab, 4c, 4d and 4e (Table S1), by indirect ELISA essentially as described [12]. Briefly, each isolate was grown overnight in BHI broth, washed, formalin killed and stored in PBS with 50% (v/v) glycerol at −20°C. Formalin-killed cells were used to coat 96 well NUNC plates (Thermo Scientific), at a concentration of 5×10^7^ cells/mL and 100 µL/well, overnight in 60 mM carbonate buffer at pH 9.6. A predetermined dilution of each MAb TCF and an irrelevant MAb against *E. coli*, used as a control, were incubated at room temperature on the plate wells for 1 hr. HRP-conjugated goat anti-mouse IgG, Fcγ fragment specific antibody (Jackson ImmunoResearch) was used to detect bound MAbs. After incubation with the substrate solution containing 0.1% (w/v) 2,2-azino-bis(3-ethylbenzthiazdine-6-sulfonic acid) and 3% (v/v) hydrogen peroxide in citrate buffer (0.02 mM citric acid, 0.03 mM tri-sodium citrate) for 10 min, the OD_414_ values were measured. Each MAb was tested in duplicate against both the positive control and the sample isolate on each plate. If the MAb reaction with the sample isolate produced an average OD_414_ value that was greater than 25% of the same MAb on the positive control isolate, it was recorded as a positive reaction; OD_414_ values below 25% of the positive control were defined as negative reactions. Three independent experiments were performed for every MAb/isolate combination.

## Results

### Identification of the Antigens

**Figure 1:**
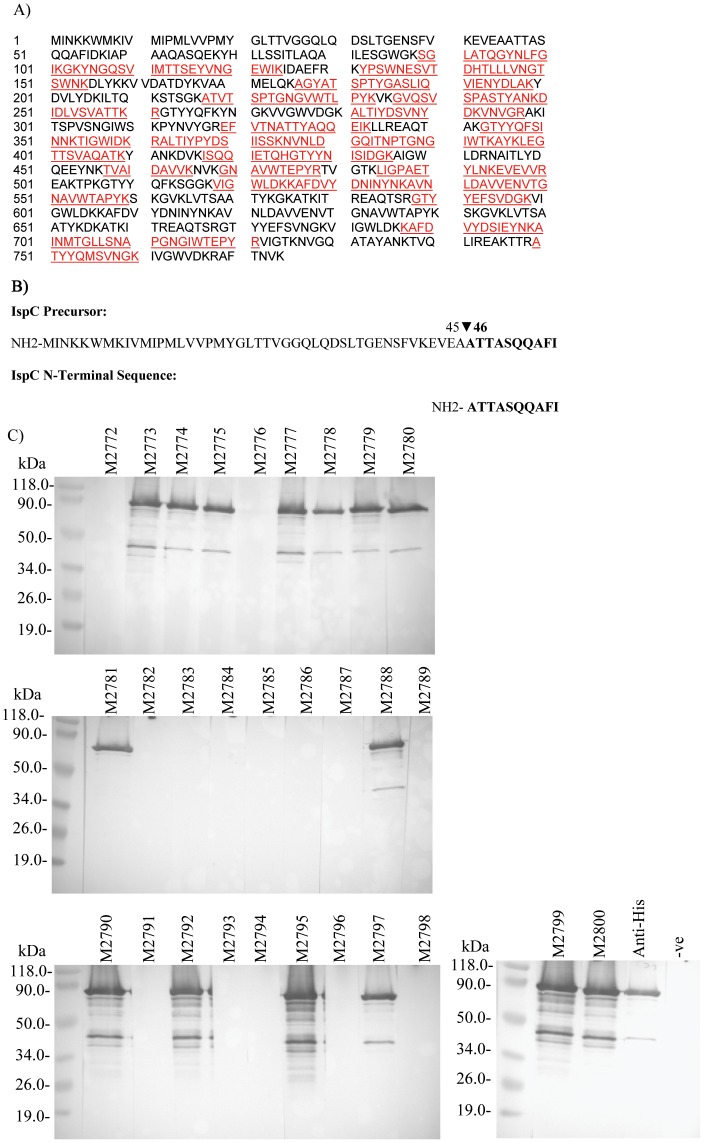
IspC is the antigen recognized by each MAb. Protein identification was performed on the protein immunoprecipitated by M2799 with MS. MASCOT software was used to match the observed MS/MS spectra against protein sequences in the NCBInr database. This analysis indicated that IspC is the most likely protein match, with a MASCOT score of 2538. Peptides which were identified by their MS/MS spectrum as matching IspC sequence are shown in red (A). Amino acid sequences shown in black were not detected during MS analysis, but are shown to illustrate the proportion of the IspC protein which was identified by MS. N-terminal sequencing of the immunoprecipitated protein yielded ten residues that aligned perfectly with residues 45–55 of the IspC protein (B). Fifteen previously generated MAbs [10] reacted strongly on a western blot with recombinant IspC (C).

The ∼77 kDa surface protein recognized by 16 MAbs (M2773, M2774, M2775, M2777, M2778, M2779, M2781, M2785, M2787, M2790, M2792, M2794, M2795, M2797, M2799 and M2800) [10] was successfully isolated from the *L. monocytogenes* surface protein extract by immunoprecipitation with MAb M2799. The ∼77 kDa protein band, resolved by SDS-PAGE after immunoprecipitation, was excized and subjected to MS analysis. The results showed that the 77 kDa antigen was IspC (Fig. 1A). N-terminal sequencing of the isolated 77 kDa antigen yielded an amino acid sequence, of 10 amino acids (AA), that aligned with AA 46 to 55 of IspC (Fig. 1B). This showed that IspC has a 45 amino acid signal sequence. Based on its deduced amino acid sequence, after cleavage of the signal sequence, the IspC protein has a molecular mass of 80.8 kDa closed to its apparent MW of 77 kDa. Each of the MAbs originally generated by Lin et al. [10] were examined for reaction with recombinant IspC (rIspC) [15]. Western blotting (Fig. 1C) revealed that 15 MAbs (M2773, M2774, M2775, M2777, M2778, M2779, M2780, M2781, M2788, M2790, M2792, M2795, M2797, M2799 and M2800) each reacted to rIspC. While M2787, M2785 and M2794 reacted with the 77 kDa native protein antigen [10] they did not react to rIspC. Although modification of the mature IspC is not known in *L. monocytogenes*, this may be hypothesized as an explanation for this different binding of the three MAbs to the IspC protein expressed between *L. monocytogenes* and *E. coli*. In contrast, M2780 and M2788 that reacted negligibly with the 77 kDa native protein [10] each showed strong reaction to rIspC. The 15 MAbs which reacted with rIspC were selected for further characterization.

### Epitiope Localization Using Recombinant IspC Fragments

**Figure 2:**
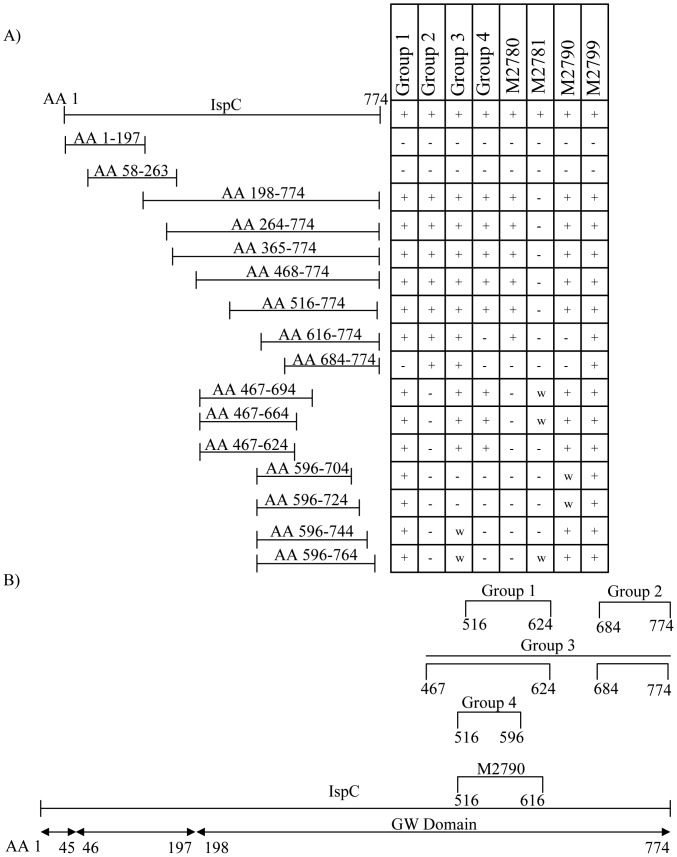
Epitopes for each MAb are localized to the C-terminal CBD of IspC. The left panel (A) provides a representation of each of the recombinant truncated IspC proteins that were produced in *E. coli*. The right panel (A) shows a summary of the ability of the corresponding truncated proteins to react with the MAbs. Group 1 consists of M2773, M2788, M2792, M2795 and M2800. Group 2 is composed of M2775 and M2797. Group 3 contains M2777 and M2778. Group 4 is composed of M2774 and M2779. The MAbs M2780, M2781, M2790 and M2799 are each in their own group since they were the only MAbs with their particular reaction profile. An illustration of the approximate location of the epitope for each MAb on the IspC protein is shown in (B).

Epitopes were mapped for 15 MAbs capable of recognizing the denatured rIspC (Fig. 1C), by constructing a series of His-tagged recombinant proteins with deletions of defined amino acid (AA) sequences of IspC (left panel, Fig. 2A). Reactivity of the recombinant protein fragments with each MAb were analyzed by western blotting and the results are summarized in Fig. 2A (right panel). The epitopes recognized by all 15 MAbs were found to be within the C-terminal cell-wall binding domain (CBD) which spans AA 198 to 774 of IspC (Fig. 2B). This was supported by the lack of reaction between each recombinant protein fragment within the N-terminal peptidoglycan hydrolase domain of IspC (AA1-197) and any of the MAbs. The epitopes for each MAb were further defined to be within the region of AA 198 to 774 by creating additional recombinant fragments, with the two exceptions: (i) all truncated fragments (with the exception of weak reactions with AA 467–694, AA 467–664 and AA596–764) were non-reactive to M2781 and (ii) M2799 which reacted with all the fragments generated with the region of AA 198 to 774.

Group 1 MAbs (M2773, M2788, M2792, M2795 and M2800) showed the same pattern of reactivity to internal fragments located between AA 197–774 of the IspC protein and recognized each, except AA 684–774 (Fig. 2A). This indicated that the epitope(s) recognized by these five MAbs was between AA 616 and AA 684. However, these MAbs also recognized an epitope within the region of AA 467 to 624, as demonstrated by antibody reactivity to the three fragments AA 467–694, AA 467–664 and AA 467–624. Reaction with fragments AA 616–774 and AA 467–624 by these five MAbs suggests that a 9 AA stretch between AA 616 and AA 624, which common to both fragments, is critical for antibody binding. However, since the CBD contains several repeated regions it is likely that the MAbs also react to similar sequences elsewhere within the CBD.

The epitope recognized by group 2 MAbs (M2775 and M2797) was mapped to a smaller C-terminal region (AA 684 to 774) of IspC. M2780 had a similar reaction profile except that while it reacted to AA 616–774 it did not react to AA 684–774. A small deletion of the last 10 C-terminal residues, as shown by the fragment AA 596–764, completely abolished the reactivity to M2775, M2797 and M2780 indicating that the last 10 C-terminal residues are critical for binding of these MAbs.

The epitopes for the group 3 MAbs, M2777 and M2778, were mapped to the region AA 684 to 764 (Fig. 2A). Serial deletions of 20 residues from the C-terminus resulted in two fragments AA 596–764, AA 596–744 which weakly reacted to M2777 and M2778. Additional C-terminal deletions created fragments AA 596–724 and AA 596–704, which did not react to M2777 and M2778. This indicates that the C-terminus is important for group 3 MAb binding. However, group 3 MAbs also showed strong reactivity to fragments AA 467–694, AA 467–664 and AA 467–624, suggesting the region between AA 467 to 624 contains a separate epitope with a similar sequence to the one contained in the region of AA 684 to 764.

Group 4 MAbs (M2774 and M2779) recognized an epitope within the AA 516–774 fragment. Further N-terminal deletions of 100 and 168 residues were made to form fragments AA 616–774 and AA 684–774, which did not react to group 4 MAbs. In addition, no reactivity to the four fragments AA 596–764, AA 596–744, AA 596–724 and AA 596–704 was observed for these two MAbs. The results indicated that the residues between AA 516 and 596 are critical to group 4 MAb binding. The reactions of these two MAbs with each of the fragments AA 467–694, AA 467–664 and AA 467–624, which each contain this hypothetical epitope, confirms this finding.

M2790 showed a reaction profile similar to that of group 1 MAbs. However, M2790 did not react with AA 616–774 indicating that the epitope is between 516 and 616. This was confirmed by antibody reactivity to the three fragments AA 467–694, AA 467–664 and AA 467–624 which all contain the AA 516–616 sequence. Reaction with the fragments AA 596–744 and AA 596–764 may indicate that the epitope is on a smaller stretch between AA 596 and 616, however, the weak reaction with AA 596–704 and AA 596–724 does not support this.

### Determination of Affinity Constants of the Monoclonal Antibodies

**Figure 3:**
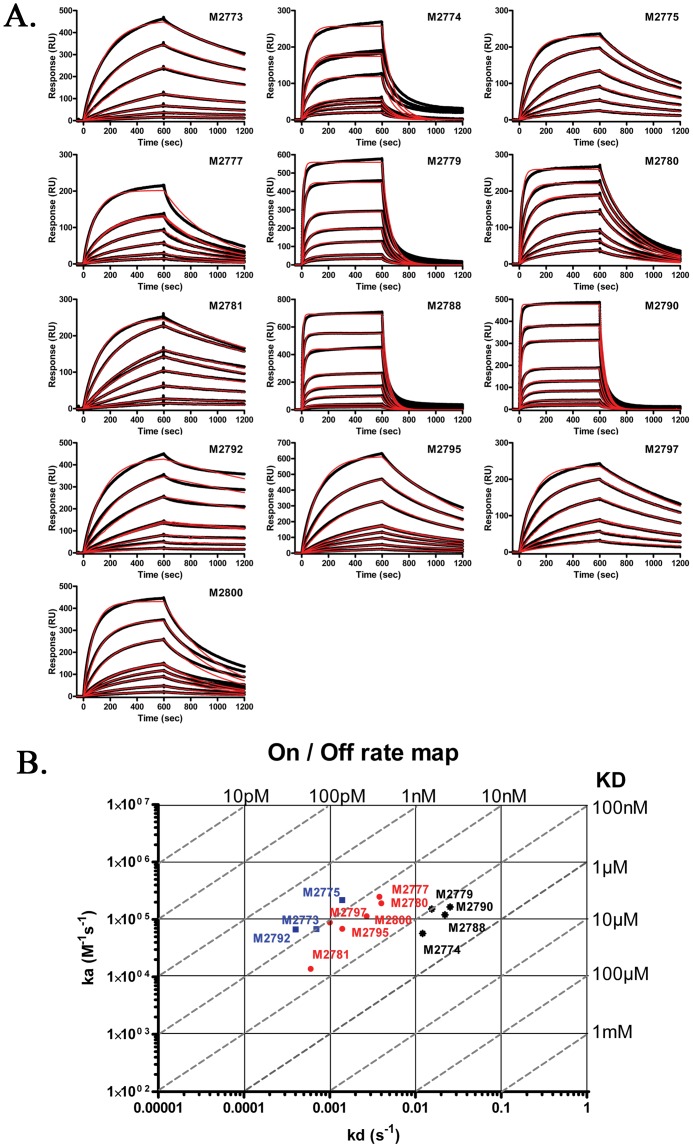
Kinetic analysis of IspC and Fab interactions. SPR sensorgrams showing FAb binding to immobilized IspC at concentrations of: 1, 2.5, 5, 10, 10, 25, 50 and 100 nM for M2773, 2.5, 5, 7.5, 10, 25, 50, 50 and 100 nM for M2774, 1, 2.5, 5, 10, 10, 25 and 50 nM for M2775, 0.5, 1, 2.5, 5, 10, 10 and 25 nM for M2777, 5, 10, 25, 50, 50, 100, 250, and 500 nM for M2779, 2.5, 5, 10, 25, 50, 50, 100 and 250 nM for M2780, 0.5, 1, 2.5, 5, 10, 10, 25 and 50 nM for M2781, 5, 10, 25, 50, 50, 100, 250, 500, and 1000 nM for M2788, 2.5, 5, 10, 25, 50, 50, 100, 250, 500 and 1000 nM for M2790, 1, 2.5, 5, 10, 10, 25, 50 and 100 nM for M2792, 1, 2.5, 5, 7.5, 10, 10, 25, 50 and 100 nM forM2795, 2.5, 5, 10, 10, 25, 50 and 100 nM for M2797, 1, 2.5, 5, 7.5, 10, 10, 25, 50 and 100 nM for M2800 are shown in (A). Black lines represent raw data measurements and red lines represent fitted curves. Rate and affinity constants are given in Table 1. A rate plane plot with iso-affinity diagonals is shown in (B). This kinetic map summarizes the respective affinities of each MAb for IspC as determined by SPR. Blue is used to denote high affinity MAbs, while red shows moderately high affinity MAbs and black is used to label lower affinity MAbs.

SPR analysis preformed on a Biacore 3000, with immobilized rIspC and Fab as analyte to determine the equilibrium dissociation constant (K_D_) of each MAb. Initial screening experiments, using only one Fab concentration, determined that M2778 had a very low affinity, so it was not selected for further SPR analysis. Separation of the Fab and Fc of M2799 by protein G chromatography proved to be difficult due to strong interactions of both the Fab and Fc with protein G. Thus, the purity of Fab required for SPR could not be achieved for MAb M2799 and it was not analyzed by SPR. For fitting of the data to a 1∶1 interaction model the analyte must have only one binding site, so the MAbs were digested with papain to produce Fabs, for the remaining 13 MAbs. SPR was preformed to determine the binding kinetics of Fabs to IspC. Different concentrations of size exclusion chromatography purified Fabs were injected over IspC, which was covalently immobilized to the dextran matrix on the sensorchip. Increase in the refractive index (caused by the accumulation of injected Fabs over immobilized IspC) was measured in real time and displayed in a sensor gram in which resonance units (RUs) are plotted against time (Fig. 3A). The K_D_, which is a measure of binding affinity ranged from 4.5 nM to 100 nM (Table 1; Fig. 3B). Five Fabs: M2773, M2775, M2781, M2792 and M2797, all have particularly high affinities. The theoretical R_max_ for IspC is 592 RU based on the formula R_max_ = (MW_analyte_/MW_ligand_) × immobilized ligand amount (RU) × stoichiometric ratio.The data showed good fitting to the 1∶1 Langmuir model, except for M2788, M2795 and M2779 which, based on RU (Table 1), likely interact with more than one site on IspC.

**Table 1:** Association rate, dissociation rate and equilibrium dissociation constants (affinities) for each monoclonal antibody.

Monoclonal Antibody	k_a_ (1/Ms)	k_d_ (1/s)	K_D_ (M)	Resonance units (RU)
M2773	5.72×10^4^	0.0007	1.2×10^−8^	506
M2774	1.16×10^5^	0.0121	1.1×10^−7^	508
M2775	2.18×10^5^	0.0014	6.4×10^−9^	266
M2777	2.48×10^5^	0.0038	1.6×10^−8^	406
M2779	1.52×10^5^	0.0155	1.0×10^−7^	613
M2780	1.92×10^5^	0.004	2.1×10^−8^	285
M2781	1.38×10^5^	0.0006	4.5×10^−9^	340
M2788	1.21×10^5^	0.0221	1.8×10^−7^	753
M2790	1.65×10^5^	0.0253	1.5×10^−7^	521
M2792	6.70×10^4^	0.0004	5.9×10^−9^	460
M2795	6.87×10^4^	0.0014	2.0×10^−8^	731
M2797	8.91×10^4^	0.0010	1.2×10^−8^	281
M2800	1.14×10^5^	0.0027	2.3×10^−8^	552

### IspC is Highly Conserved in Serotype 4b Strains

Indirect ELISA was performed to assess MAbs for cross-reactivity with different *L. monocytogenes* serotypes (Table 2). The cross-reactivity of different isolates with each MAb was calculated as a percent of the OD_414_ reading seen for that MAb and *L. monocytogenes* serotype 4b strain LI0521. Most MAbs reacted very strongly to each of the 9 *L. monocytogenes* serotype 4b isolates tested, with many reactions exceeding 100%. This indicates that IspC is conserved in serotype 4b strains. A cross-reaction was defined as any reaction with a non-serotype 4b isolate that exceeds 25% of the OD_414_ that would be seen between the particular antibody being tested and *L. monocytogenes* serotype 4b strain LI0521.

**Table 2:** Cross-reactions of anti-IspC MAbs with other *L. monocytogenes* serotypes.

	Numberof isolates screened	Number of Positive Reactions^b^ :														
*L. monocytogenes* serotypes^a^		M2773	M2774	M2775	M2777^c^	M2778	M2779	M2790	M2781^c^	M2788	M2790	M2792^c^	M2795^c^	M2797^c^	M2799^c^	M2800
1/2a	9	0	0	0	0	0	0	0	0	0	0	0	2	0	4	0
1/2b	8	0	0	0	6	0	0	0	1	0	0	1	5	0	6	0
1/2c	5	0	0	0	0	0	0	0	0	0	0	0	0	0	1	0
3a	5	0	0	0	0	0	0	0	0	0	0	0	0	0	0	0
3b	2	0	0	0	0	0	0	0	0	0	0	0	0	0	0	0
3c	1	0	0	0	0	0	0	0	0	0	0	0	0	0	0	0
4a	2	2	0	0	1	1	1	0	2	2	0	2	2	0	2	2
4b	9	9	8	9	9	9	9	9	9	9	8	9	9	9	9	9
4ab	2	2	2	2	2	2	2	2	2	2	2	2	2	2	2	2
4c	4	4	0	0	1	0	1	0	4	2	0	4	4	0	4	4
4d	2	0	0	0	0	0	0	0	0	0	0	0	0	0	0	0
4e	1	0	0	0	0	0	0	0	0	0	0	0	0	0	0	0

a Isolate details can be found in Table S1.

b The interaction between each antibody-isolate pair was examined using ELISA in three independent experiments. Positive reactions were recorded if the average of the three OD_414_ measurements was >25% of the OD_414_ recorded for the same antibody when reacting with *L. monocytogenes* serotype 4b strain LI0521.

c Negative reactions are reported if the average OD_414_ of the three independent experiments was <25% of the OD_414_ recorded for the same antibody when reacting with *L. monocytogenes* serotype 4b strain LI0521. In most cases a negative reaction could also be defined by OD_414_ of all three experiments being below the 25% threshold. However, M2777, M2781, M2792, M2795, M2797 and M2799 each frequently had one or two of three the measurements above the 25% threshold, even though the average remained below 25%. The variability of each of these MAbs makes them poor candidates for diagnostics.

The MAbs M2795 and M2799 were able to detect 2 and 4 of 9 *L. monocytogenes* serotype 1/2a isolates, respectively. In addition, M2777, M2781, M2792, M2795 and M2799 were able to detect 6, 1, 1, 5 and 6 of 8 *L. monocytogenes* serotype 1/2b isolates, respectively. Several cross-reactions occurred between serotype 4a and 4c isolates. However, M2774, M2775, M2780, M2790 and M2797 did not react with any serotype 4c or serotype 4a isolates. These five antibodies were specific for serotype 4b and of the 41 non-serotype 4b isolates tested only cross-reacted with serotype 4ab isolates. BLASTp revealed that a few annotated hypothetical proteins had homology with IspC. LMIV_0809, a putative protein from *L. monocytogenes* serotype 4a FSL J1-208, showed 95% homology with IspC. The CBD of IspC also shares 89% identity with lin1064 and 90% identity with lwe1056, hypothetical proteins from *L. innocua* and *L. welshimeri*, respectively. This may explain why some MAbs cross-reacted with these closely related serotypes.

## Discussion

This study characterized 15 MAbs and demonstrated that each recognizes linear epitopes located within the CBD of IspC. IspC is a cell wall associated protein that has *N*-acetylglucosaminidase activity [11]. IspC was previously shown to be involved in virulence [16], and in this work we have shown it to be well conserved within the 4b serotype. Several of the anti-IspC MAbs did not react with any of the other *L. monocytogenes* serotypes tested providing evidence that IspC is exclusive to the *L. monocytogenes* serotype 4b, and may have a role in the high degree of virulence of this serotype. This study is unique because it provides the most extensive characterization of MAbs produced against *L. monocytogenes* published to date. In addition, the novel MAbs described in this work have potential to be used as reagents in future research aimed at elucidating the role of IspC in virulence.

The 15 MAbs that recognize IspC were generated by immunizing mice with formalin killed *L. monocytogenes* serotype 4b whole cells [10]. This immunization strategy produced a total of 23 MAbs that interacted with the *L. monocytogenes* cell surface; however, only 16 detected a protein band in a western blot of total cellular proteins [10]. We provide conclusive evidence that 15 MAbs recognize rIspC. Thus, immunization with formalin killed whole cells created a group of MAbs, most of which target the same surface protein despite other possible surface antigens being readily available. An explanation may be that IspC is an immuno-dominant surface antigen of *L. monocytogenes* serotype 4b. This interpretation is supported by previous findings that IspC is a primary target of the humoral immune response to *L. monocytogenes* serotype 4b infection [17]. Previous studies aiming to develop MAbs against *L. monocytogenes* serotype 4b as diagnostic reagents have not always determined the molecular identity of antigens being targeted by MAbs; thus it is unclear if any were in fact against IspC. An earlier study that used *L. monocytogenes* whole cell lysate as an immunogen generated only 2 MAbs which recognized cell surface localized antigens, although, a total of 35 MAbs were produced [12]. The 2 MAbs that recognized surface antigens did not detect protein bands in western blots of total cellular proteins, indicating that the MAbs either recognized conformational epitopes or that the target antigen was non-protein in nature [12]. Another study that used formalin killed whole cells for immunization generated MAbs that recognized cell-surface antigens and were highly specific for *L. monocytogenes* serotype 4b [9]. However, inconsistent western blot results led to failure to provide an estimate of the MW of the antigen [9]. Accurate predictions as to whether the MAbs from either of these studies also recognize IspC are impossible. Establishing the molecular identity of the antigen as IspC is extremely important for the full assessment of its value as a diagnostic marker. This findings has motivated us to characterize the IspC expression with respect to its diagnostic suitability in a recent study [18] leading to identification of the transcription start site and a functional promoter of the *ispC* gene and in-depth elucidation of the protein expression under various environmental conditions relevant to those encountered by the pathogen in food samples. This, together with the data reported here, has established the basis for IspC to be targeted for the pathogen detection and/or capture with anti-IspC MAbs.

Epitope localization experiments demonstrated that all the MAbs investigated in this study recognize sequences within the C-terminal CBD of the IspC protein. IspC has a modular domain structure with an N-terminal signal sequence (AA 1–45), a peptidoglycan (PG) hydrolase domain (AA 46–197) and a C-terminal CBD (AA 198–774) (Fig. 2B) [15]. N-terminal sequencing showed that IspC contains a 45 AA N-terminal signal peptide which is cleaved prior to cell wall targeting. This is in contrast to earlier findings that IspC has an N-terminal signal sequence of 23 AA when produced in *E. coli* [19]. The differences in signal peptide processing between *L. monocytogenes* and *E. coli* are not surprising, since the signal sequence of secreted proteins are generally longer in Gram-positive bacteria than in Gram-negative bacteria [20]. The CBD of IspC is composed of 7 tandem repeats called GW-modules. These GW-modules are responsible for anchoring the protein to the cell wall via a non-covalent interactions with lipotechoic acids (LTA) [21]. Previous work has demonstrated that the anti-IspC MAbs investigated in this study interact with the cell surface of live cells [10]. The epitope mapping findings presented in this work, support a model where GW-modules are exposed at the cell-surface and not embedded in the PG. Analysis of the IspC amino acid sequence provides additional support for this model since the probability of cell surface exposure is quite high for stretches of the CBD based on an Emini plot [22]. Additional studies also suggest that the GW-modules are surface exposed. Antibodies produced specifically against GW-modules are protective against *Erysipelothrix rhusiopathiae* [23]. For antibody protection GW-modules would have to be cell-surface exposed and accessible to the antibodies.

**Figure 4:**
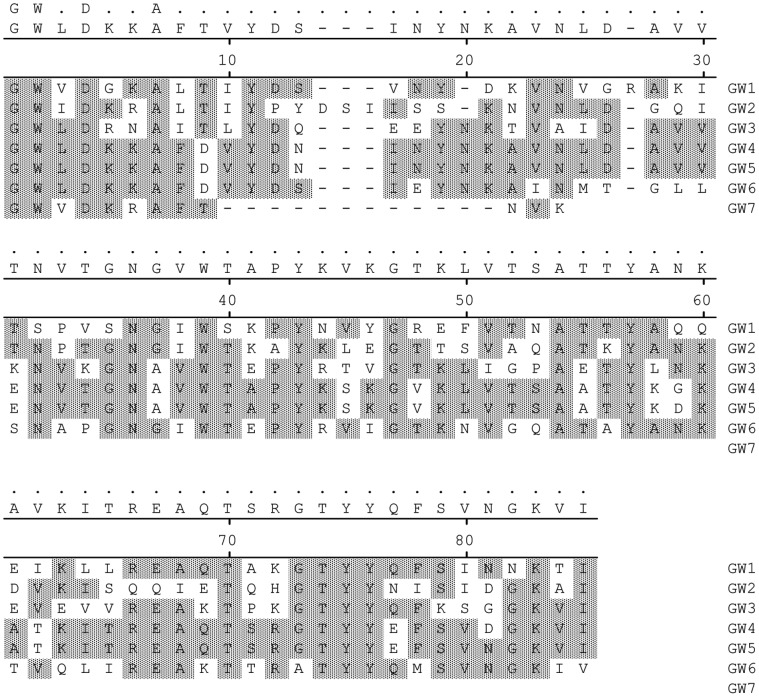
Sequential GW-modules in the CBD of IspC have areas of significant sequence homology. Matching residues are shaded.

The repetitive nature of the CBD complicated the interpretation of the epitope localization experiments in this study since the homology of the GW-modules made it possible for the MAbs to have multiple epitopes within the same protein (Fig. 4). The RU values reported for M2779, M2788, and M2795 exceeded the theoretical R_max_ (Table 1) indicating multiple epitopes for these 3 MAbs. The high RU observed for other MAbs, although not exceeding R_max_, combined with the observed reactions with truncated IspC fragments may also indicate multiple epitopes for additional MAbs.

Low affinity or avidity has been cited as a reason that antibody based protocols, such as immunomagnetic separation or flow cytometry, fail to detect *L. monocytogenes* cells [24]. We carried out a full kinetic analysis of the interaction between each MAb and IspC to select the optimal MAb for future diagnostic purposes. This is the first study to determine the affinity of MAbs against *L. monocytogenes*. High affinity is a requirement of diagnostic tests, such as immunomagnetic separation or flow cytometry [25]. The affinities of the MAbs from this study, in the nanomolar range, are within the biologically useful range for current diagnostic tests such as ELISA and M2773, M2775, M2781, M2792 and M2797 have a particularly high affinity compared to other MAbs produced using a similar methodology [26], [27], [28]. In a clinical setting, high-affinity MAbs have a greater neutralizing potential than low-affinity MAbs during passive immunization [29]. Future work with these selected MAbs against *L. monocytogenes* serotype 4b will concentrate on the ability of these MAbs to diagnose this important serotype using novel, culture independent, diagnostic platforms currently under development by our group.

Certain anti-IspC MAbs also have a high specificity for *L. monocytogenes* serotype 4b, particularly M2774, M2775, M2780, M2790 and M2797 which showed the most fidelity to *L. monocytogenes* serotype 4b and of the 41 non-serotype 4b isolates tested, did not react with any except for those identified as serotype 4ab. Cross-reactions with serotype 4ab isolates may be expected since the *Listeria* serotyping scheme is based on positive reactions between polyclonal antiserum raised against somatic and flagella antigens [30]. By definition serotype 4ab isolates contain the combined somatic antigens of both serotype 4a and 4b. Given that IspC is a cell wall-associated protein (i.e., somatic antigen), it is not at all surprising to observe that these MAbs recognizing the IspC of serotype 4b isolates also cross react with serotype 4ab isolates.

The CBD of IspC shares some homology with the GW-modules of Ami 4b [31] and InlB [19]. Ami 4b is also unique to *L. monocytogenes* serotype 4b and cross-reactions of the MAbs with this protein would not be detected by our methodology. The observed homology with InlB conflicts with the low cross-reaction observed between anti-IspC MAbs and other commonly pathogenic *L. monocytogenes* serotypes, such as 1/2a and 1/2b, since InlB is part of the prfA regulon and conserved in pathogenic isolates. Low cross-reactions between anti-IspC MAbs and InlB containing serotypes could indicate that homology within the epitope is not considerable enough, or that the epitope is not surface exposed, or that InlB is expressed poorly when cells are grown in BHI broth. Interestingly, GW-modules have been suggested for inclusion in vaccine preparations since the homology among GW-modules from different organisms would allow for cross-protection against related pathogens [32]. In contrast, the MAbs against IspC GW-molecules show little cross-reaction with related isolates therefore MAb interaction is likely limited to the IspC protein.

Several attempts have been made to generate *L. monocytogenes* specific antibodies by raising MAbs against the main virulence factors (InlA, InlB, LLO, ActA), however, most of these MAbs have failed in diagnostics since the targets are not expressed well during *in vitro* growth conditions [33]. This study presents the first MAbs known to date that specifically recognize *L. monocytogenes* serotype 4b and are therefore excellent candidates for use in the detection and/or capture of this clinically important serotype. In addition, since IspC is unique to *L. monocytogenes* serotype 4b, its contribution to the highly pathogenic phenotype of this serotype should be investigated in future studies.

## Supporting Information

Table S1 *Listeria monocytogenes* isolates used in this study. a- Indicates the strain used in immunization during the production of the MAbs.
(DOC)

Table S2 Primers used to amplify fragments coding for possible IspC epitopes that react with the MAbs. a – NdeI restriction site is shown underlined. b – NotI restriction site is shown underlined.
(DOC)
