# Supplementary material for: Monoclonal Antibodies Recognizing the Surface Autolysin IspC of Listeria monocytogenes Serotype 4b: Epitope Localization, Kinetic Characterization, and Cross-Reaction Studies
Source: PLoS One. 2013 Feb 4;8(2):e55098. doi: 10.1371/journal.pone.0055098 (PMC3563664; doi:10.1371/journal.pone.0055098)
Supplement: Table S1 — Listeria monocytogenes isolates used in this study. a- Indicates the strain used in immunization during the production of the MAbs. (DOC) [file pone.0055098.s001.doc]

| ***L. monocytogenes* Isolate ID Number** | **Serotype** |
| --- | --- |
| HPB 5327 | 1/2a |
| HPB 4705 | 1/2a |
| OLF 09016 | 1/2a |
| OLF 09033 | 1/2a |
| OLF 09011 | 1/2a |
| OLF 09049 | 1/2a |
| OLF 09015 | 1/2a |
| HPB 6036 | 1/2a |
| HPB 6095 | 1/2a |
| HPB 5330 | 1/2b |
| HPB 4857 | 1/2b |
| OLF 090271 | 1/2b |
| OLF 09060 | 1/2b |
| OLF 09040-1 | 1/2b |
| HPB 5328 | 1/2b |
| HPB 5913 | 1/2b |
| HPB 6027 | 1/2b |
| HPB1869 | 1/2c |
| HPB 5121 | 1/2c |
| OLF 09013 | 1/2c |
| OLF 09022-1 | 1/2c |
| HPB 2972 | 1/2c |
| HPB 2768 | 3a |
| HPB 3058 | 3a |
| OLF 09005 | 3a |
| OLF 09039 | 3a |
| HPB 5665 | 3a |
| HPB 4909 | 3b |
| HPB 1031 | 3b |
| HPB 61 | 3c |
| HPB 3501 | 4a |
| HPB 5041 | 4a |
| HPB 5058 | 4b |
| LI0521 | 4b *^a^* |
| HPB 3449 | 4b |
| HPB 5251 | 4b |
| HPB 5364 | 4b |
| HPB 5816 | 4b |
| HPB 5906 | 4b |
| HPB 6024 | 4b |
| HPB 6092 | 4b |
| HPB 1848 | 4b |
| HPB 520 | 4ab |
| HPB 1265 | 4ab |
| HPB 5248 | 4c |
| HPB 4497 | 4c |
| HPB 4706 | 4c |
| HPB 5248 | 4c |
| HPB 18 | 4d |
| HPB 4534 | 4d |
| HPB 1861 | 4e |

1. Indicates the strain used in immunization during the production of the MAbs.
