# Supplementary material for: Monoclonal Antibodies Recognizing the Surface Autolysin IspC of Listeria monocytogenes Serotype 4b: Epitope Localization, Kinetic Characterization, and Cross-Reaction Studies
Source: PLoS One. 2013 Feb 4;8(2):e55098. doi: 10.1371/journal.pone.0055098 (PMC3563664; doi:10.1371/journal.pone.0055098)
Supplement: Table S2 — Primers used to amplify fragments coding for possible IspC epitopes that react with the MAbs. a – NdeI restriction site is shown underlined. b – NotI restriction site is shown underlined. (DOC) [file pone.0055098.s002.doc]

| Primer | Nucleotide Sequence | Used to Amplify (Amino Acids) | Position (Nucleotides) |
| --- | --- | --- | --- |
| Lin822F | 5’- acacatatgatctatccgtatgattcc -3’ *^a^* | 365-774 | 1093-1110 |
| Lin824F | 5’- acacatatgaagggtaatgctgtatggaca -3’ *^a^* | 468-774 | 1402-1422 |
| Lin825F | 5’- acacatatgggcaaagtaatcggctggtta -3’ *^a^* | 516-774 | 1546-1566 |
| Lin827F | 5’- acacatatgtacaacaaagcggttaac -3’ *^a^* | 616-774 | 1846-1863 |
| Lin829F | 5’- acacatatgttagataaaaaagcttttgat -3’ *^a^* | 684-774 | 2050-2070 |
| Lin830R | 5’- gtggcggccgctttaacgtttgtaaaagctc -3’ *^b^* | multiple | 2303-2322 |
| Lin837F | 5’- ACACATATGGTGAAGGGTAATGCTGTATG -3’ *^a^* | multiple | 1399-1418 |
| Lin836F | 5’- ACACATATGGATGGTAAAGTCATTGGCTG -3’ *^a^* | multiple | 1786-1805 |
| Lin845R | 5’- GTGGCGGCCGCAGAATCATATACATCAAAAGC -3’ *^b^* | 467-694 | 2062-2082 |
| Lin846R | 5’- GTGGCGGCCGCCGCTTCTCGAGTTATCTTAG -3’ *^b^* | 467-664 | 1973-1992 |
| Lin847R | 5’- GTGGCGGCCGCAGCATCTAAGTTAACCGCTT -3’ *^b^* | 467-624 | 1853-1872 |
| Lin848R | 5’- GTGGCGGCCGCAGTCATATTAATCGCTTTAT -3’ *^b^* | 596-704 | 2093-2112 |
| Lin849R | 5’- GTGGCGGCCGCGCCAATAACTCTATACGGCT -3’ *^b^* | 596-724 | 2153-2172 |
| Lin850R | 5’- GTGGCGGCCGCCTCGCGTATCAACTGTACTG -3’ *^b^* | 596-744 | 2213-2232 |
| Lin851R | 5’- GTGGCGGCCGCCCAACCAACTATTTTACCAT -3’ *^b^* | 596-764 | 2273-2292 |

a – NdeI restriction site is shown underlined.

b – NotI restriction site is shown underlined.
